# Towards greener and more sustainable pre-clinical oncology research

**DOI:** 10.1038/s44276-024-00115-0

**Published:** 2025-01-22

**Authors:** Cian Campion, Linda Robertson, Ian Stansfield, Valerie Speirs

**Affiliations:** 1 School of Medicine, Medical Sciences and Nutrition, University of Aberdeen, Aberdeen, UK; 2 Aberdeen Cancer Centre, University of Aberdeen, Foresterhill, Aberdeen, AB25 2ZD UK

## Abstract

Single-use plastics (SUPs) are used widely in cancer research laboratories. They are cheap, durable, and lightweight, and until now have been considered disposable items. This, however, contributes significantly to unsustainable waste production. SUP waste is typically diverted to landfill or incineration, which contributes to greenhouse gas emissions, taking many years to degrade. Lack of robust SUP waste disposal streams, particularly in cancer research labs has long term effects on the environment. Having identified that a single laboratory researcher in our group generates at least 15 kg SUP tissue culture waste alone each year, we explore some of the issues associated with SUPs in pre-clinical oncology research, discussing potential reuse routes, alternative materials for labware and developing circular approaches to plastic consumption to address the green agenda. We also propose recommendations for improving sustainability in cancer research labs.

## Introduction

Plastic stands as a fundamental component of modernisation. Permeating every aspect of human existence, its unparalleled stance as a versatile, durable and cost-effective material has propelled its industrial potential with global demand across all sectors. A single study of 350 researchers from the University of Exeter, UK, estimated that 5.5 million tonnes of plastic waste were generated each year from biomedical research laboratories [1]. The daily use of disposable single-use plastics (SUPs) contributes significantly, with their production and eventual disposal having a detrimental impact on the environment [2]. This extends to cancer research laboratories where there has been a strong reliance on SUPs over the last 3 decades. SUPs are used in the manufacture of a range of labware, including pre-sterilised tissue culture flasks, multi-well plates, Petri dishes, Stripettes, pipette tips to name but a few, which are all mainstays of preclinical in vitro studies in cancer research. Indeed, one of us (VS) recalls the transition from glass to plastic pipettes in the early 1990s happened almost overnight. This was embraced by the community as plastic was regarded as a more robust, safer and lightweight alternative to glass and resulted in cancer research laboratories transitioning to almost 100% SUP consumables. Their convenience, supplied in a sterile, ready to use format, is countered by the creation of considerable waste. Motivated to discover the volume of SUP waste generated in our research group from tissue culture experiments alone, we audited 3 postgraduate and 2 undergraduate project students over a week. Collectively they generated 1874g i.e. 375 g per student, of which 60% was polystyrene (Table 1). Assuming a typical year is 40 working weeks, this equates to at least 15 kg SUP tissue culture waste generated each year by a single laboratory researcher in our group. Of course, this does not account for SUP waste generated by other common laboratory procedures e.g. qPCR so the total amount of SUP waste will be significantly higher. Furthermore, much SUP waste generated by laboratories is still bagged and autoclaved, itself an energy-hungry process, through the electricity required to generate high-temperature, pressurised steam. Autoclaved products may then end up in landfill where they take several generations to degrade.

**Table 1:** Type and volume of SUP waste generated by five students from tissue culture experiments in a typical working week.

Tissue culture SUP (weight, g)^1^	Quantity	Total weight SUP (g)^2^
Flasks		
T25 (16.5); T75^50^	7, 7	466
Eppendorf tubes		
0.5 ml (0.5); 1.5 ml (1)	4, 3	5
Falcon tubes		
15 ml (6.5); 50 ml (13)	11, 11	215
Stripettes^a^		
5 ml (9.5); 25 ml (10.5)	72, 1	695
Pipette tips		
P1000 (0.55); P200 (0.28)	17, 196	64
Multi-well plates^a^		
6-well (59); 12-well (64.5); 96-well (63.5)	2, 2, 1	311
Autoclave bags (35.5)	5	118
**SUP waste generated**		**1874g**

SUP waste generated by each student was collected, counted, weighed and categorised into type. Suppliers are listed in Table 2.

^a^Includes weight of wrapping.

^1^Absolute weight

^2^Weight rounded up.

But times have changed. SUP waste is a significant environmental issue [2], with estimates that throughout their lifecycle, including production and disposal, all global plastics contribute 1.8 billion tonnes of greenhouse gases [3]. The UK government announced bans on a range of household SUPs in all four UK nations. This policy was implemented in Scotland & Northern Ireland in 2022 [4, 5] and in England & Wales in 2023 [6]. Subsequent reductions in SUPs were realised in the food and drink industries and household recycling schemes have reduced the volume of SUP waste which ends up in landfill. Cancer research laboratories must also adapt to play their part in reducing carbon emissions.

## Sustainability benchmarks for cancer research laboratories

Greener solutions for laboratories exist, notably the Laboratory Efficiency Assessment Framework (LEAF) Project developed by University College London [7]. LEAF is an online metric designed to help labs identify how they can transition to net-zero. It provides a set of criteria for reducing SUP amongst other sustainable actions and then allows users, which can range from a single lab group to an entire research institute, to assess how they stand regarding sustainability and track their improvements as they implement changes. Gold, Silver or Bronze status is then given, benchmarked against how well each sustainability criterium is implemented. To assess its efficacy, LEAF was piloted across 235 laboratory groups from 23 universities or research institutes in the UK and Ireland between 2018 and 2020. As well as making savings, on average £3700 annually with total savings exceeding £640,000, 648 tCO_2_e were avoided and 99% of users indicated that they would use LEAF again [8]. LEAF is not restricted to the UK; in 2024, 85 organisations worldwide had signed up to the programme. Other green lab solutions exist e.g. the UK Laboratory Efficiency Action Network [8], with further networks documented across Europe and North America [9].

## Barriers and potential solutions to implementing sustainability in cancer research laboratories

Scientific training mandates strict adherence to tried and tested standard operating procedures, underscored by the current focus on scientific reproducibility in cancer research [10, 11]. This creates in researchers a natural reluctance to disrupt the status quo in their lab working practices simply to increase sustainability. Cancer research scientists typically use considerable amounts of SUP in their daily work, which are frequently used once to avoid inter-experimental cross-contamination. Only a tiny fraction of this is recycled, with differences in recycling behaviour clearly recognised between the home and the workplace, where awareness of recycling streams for the latter may not be as obvious as the former [12, 13].

Early Career Researchers working on fixed term contracts often feel pressured to sustain research progress, hence may have perceptions that incorporating sustainability considerations into their workflow will come at a cost to them personally, through sacrifice of time, quality, and career progression and with no apparent direct benefits to them, especially in a climate where competition for research funding is strong. Moreover, there are understandable concerns about recycling SUPs originating from cancer research laboratories and indeed laboratories in general as there are perceptions that these may be contaminated with hazardous substances, which is certainly true in some cases. Typical SUP items used in cell culture experiments in cancer research laboratories like ours are listed in Table 2.

**Table 2:** SUP items commonly used for cell culture experiments in cancer research laboratories.

SUP	SUP type	Recyclable	Supplier (catalogue number)
**Liquid handling**			
Stripettes part plastic wrap (5 ml, 10 ml, 25 ml)	PS / EVA^1^	Yes^2^	Fisher (5 ml, 10420201; 10 ml, 10084450; 25 ml, 1060615)
Stripettes all plastic wrap (5 ml, 10 ml, 25 ml)	PS^3^	Yes^2^	Fisher (5 ml, 11829660; 10 ml,11839660; 25 ml, 11517752)
Pipette tips (P10, P200, P1000)	PP	Yes^2^	Starlab (P10, S1111-3700; P200, S1111-1706; P1000, S1111-2721)
**Tissue culture vessels**			
Flasks (T25, T75)	PS	Yes^2^	Greiner (T25, 658175; T75, 660175)
Petri dishes (35 mm, 100 mm diameter)	PS	Yes^2^	Greiner (35 mm, 627160; 100 mm, 664160)
Multi-well plates (6, 12, 24, 48, 96 well)	PP	Yes^2^	Nunc (6-well Nunc; 1011983) Corning (12-well ;10253041 48- well; 10732552) Greiner (24-well, 662160; 96-well, 655180)
**Containers for tissue culture solutions**			
Tissue culture medium	PET	Yes	Sigma (RPMI 1640, R8758-500ML) Life Technologies (DMEM GlutaMAX, Gibco 11594446)
Phosphate-buffered saline	PET	Yes	Sigma (P4417)
Foetal bovine serum	PET	Yes	Biosera (FB-1092)
Trypsin	PET	Yes	Sigma (T3924-500ML)
**Tubes**			
Eppendorf tubes (0.2, 0.5, 1.5 ml)	PP	Yes^2^	Fisher (0.2 ml, 1178110) Axygen (0.5 ml, MCT-060-C; 1.5 ml MCT-175-C)
Universal (30 ml)	PP	Yes^2^	Sterilin (12521299)
Bijou (5 ml)	PS	Yes^2^	Sterilin (11369123)
Cryovial (1.8 ml)	PP	Yes^2^	Starlab (E3110-6122)
**Miscellaneous**			
Autoclave bags	PP	Yes	Fisher (11309103)
Product outer packaging	Various	Mostly	Multiple

*PS* Polystyrene, *PP* Polypropylene, *PE* Polyethylene, *PET* Polyethylene terephthalate, *EVA* Ethylene Vinyl Acetate.

^1^Outer wrapper is part PS, part EVA and requires separation into different recycling streams.

^2^Initially sterile but treated as hazardous waste after being in contact with cancer cells.

^3^Recycling costs are higher than for other SUPs [30].

Since forward planning of experiments is routine, researchers could consider incorporating pre-experimental environmental audits into their workflows prior to starting experiments or establishing new protocols. This could take account of, for example; size and type of plates/tubes used; using Stripettes wrapped in all-plastic wrap which is easier to recycle than plastic/EVA wrap; considering if reusable glass tubes could be used instead of SUP; asking if there are points in the experiment where Stripettes/pipette tips/gloves could be used more than once; considering if a SUP lab item made with plastic that is easy to recycle could substitute for one made from a non-recyclable SUP etc. By doing this, scientists themselves can be the true proponents of change by advocating sustainable science through behavioural change [14].

Currently, following autoclaving with other non-plastic biohazard waste, SUP waste generated from our labs is sent for incineration. Resulting ash is recycled, most commonly into building materials. Energy is recovered as a byproduct of the incineration process. As research laboratories typically use up to 5 times more energy than like-for-like commercial space [15] other easy wins to reduce energy demands could be implemented. These include ensuring non-essential laboratory equipment and computers are switched off at the end of each working day and raising the temperature set‑point on ultra-low temperature freezers from −80 °C to −70 °C. These may seem obvious but can often be overlooked.

## Creating circular economies for cancer research labs

Broadly speaking, a circular economy is defined as a symbiotic network of reusability, focusing on reducing waste generation and increasing sustainability by reusing resources and materials. There are examples of household SUPs being used in circular economies e.g. designers from Gray’s School of Art at Robert Gordon University established a city centre community recycling hub in Aberdeen, Scotland, collecting locally generated SUP waste which is recycled into plant pots, and soap dishes [16].

As discussed earlier, SUPs from cancer research laboratories are often in contact with toxic chemicals and other hazardous materials. Decontamination steps such as disinfection with biocides are required for biosafety, to remove organisms including MRSA, HIV, Hepatitis B and Herpes viruses and to kill any remaining cancer cells. Autoclaving is also necessary as SUPs can be diverted to landfill. However, there are instances where some consumables remain completely sterile e.g. tissue culture experiments, used regularly in cancer research are always set up under aseptic conditions using sterile plastic Stripettes and with sterile solutions (tissue culture medium, phosphate-buffered saline). Many of these items never come into direct contact with cancer cells yet are treated as contaminated waste. Since most academic research is done in universities which have teaching labs for undergraduate students, with careful thought and forward planning, uncontaminated SUPs such as these could be washed and repurposed to use in teaching labs. This resonates with the 6Rs approach; Review, Reduce, Reuse, Refill, Replace, Recycle, pioneered by the University of Manchester, UK which aimed to reduce the volume of SUPs used, often just once and fleetingly, in undergraduate practical classes without affecting the student experience and their learning outcomes [17]. By reviewing its protocol for plastic use, the University of Manchester managed to reduce its SUP waste, find ways of using recyclable materials, reusing and refilling labware and replacing plastics with other, more sustainably managed materials. Initial estimates included savings of 37,000 plastic items across 12 undergraduate practical classes, with a financial saving of around £3000 [18]. Other universities have reported cost savings by implementing similar strategies [19, 20].

But where does this leave hazardous SUP which forms most of the SUP waste generated by cancer research laboratories. Recently circular economies that cater for the type of SUP waste originating from Safety Level 1 and 2 bioscience laboratories have been developed. LabCycle [21] a UK company based at the University of Bath, has developed a pipeline where SUPs are sorted into defined material streams and decontaminated. These can then be recycled into pellets which are used to make new lab consumables. As a patented process, the specifics are proprietary, but the potential for cancer research laboratories to participate in schemes like this could be considerable as we move towards net zero emissions.

## Alternatives to SUPs

**Fig. 1:**
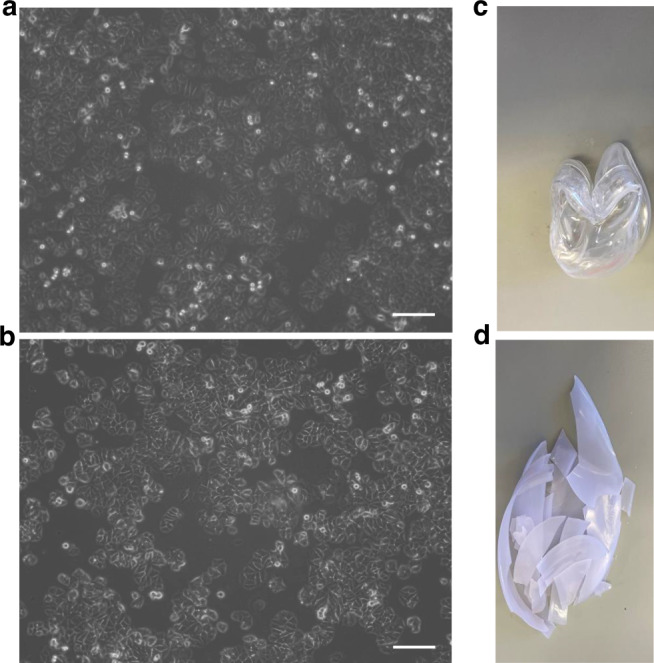
Impact of conventional SUP Petri dishes with those made from PLA on adhesion and growth of MCF-7 cells and the effects of autoclaving. Equivalent numbers of MCF-7 cells were plated onto SUP Petri dishes (Greiner; **a**) or PLA Petri dishes (Planet-Safe®; **b**) in RPMI 1640 supplemented with 5% FBS (suppliers listed in Table 1). Cell adherence and growth after 3 days in culture appeared identical. One experimental example from a series of 3 is illustrated. After autoclaving the SUP dish formed a dense solid mass **c** while the PLA one was brittle and broke into fragments **d**. Scale bar = 500 μm.

Polylactic acid (PLA) is a biodegradable and compostable thermoplastic monomer derived from renewable, organic sources such as corn starch or sugar cane [22] and has been used as an EN13432-certifed compostable packaging alternative to bioplastics, notably in the food and drink industry. Known in this sector as Vegware, it is used to manufacture a range of compostable catering disposables for hot and cold food and drinks [23]. Sustainable labware made from PLA, including Petri dishes and 96-well flat bottom plates, is now available from Planet-Safe® [24]. Proof-of principle work from our group comparing growth and morphology of MCF-7 breast cancer cells cultured on PLA versus standard plastic Petri dishes under identical conditions over 3 days showed very similar outcomes (Fig. 1). Planet-Safe® PLA dishes still need to be disposed of responsibly and become brittle when autoclaved (Fig. 1). This makes them more susceptible to degradation and composting in a matter of 2-3 months, in contrast to oil-based SUPs where this process takes decades [25–27]. The development of PLA-based lab products encouraging, and it seems likely that additional PLA-based single-use laboratory products will be developed in the near future. Currently they are 4x more expensive than conventional laboratory SUP but as demand increases the price will almost certainly drop. Other labware manufacturers are taking positive steps towards sustainability e.g. through reducing plastic packaging or replacing this with biodegradable packaging and taking advantage of take-back schemes for recycling pipette tip boxes and Styrofoam boxes.

## Conclusions and recommendations

SUPs have revolutionised in vitro pre-clinical oncology research, but this has come at a cost in terms of environmental consequences. While many cancer research groups/laboratories/Institutes have implemented recycling schemes, often these tend not to be embraced in the same way as household recycling for the reasons we have outlined here. However, positive steps are being taken by Cancer Research UK, the world’s leading cancer charity, through implementation of plan to achieve net zero by 2050 across all aspects of its business, including laboratories, some of which have already achieved LEAF Silver accreditation with plans to achieve LEAF Gold underway [28]. Based on our findings, we propose six recommendations for improving sustainability in cancer research laboratories (Table 3).

**Table 3:** Six recommendations for improving sustainability in cancer research laboratories.

1	Review and reconsider the standard methodology for SUP use within labs, communicate with other lab groups more frequently to identify a more defined pattern of waste management
2	When ordering, prioritise options with reduced or biodegradable plastic packaging
3	Where financially and methodologically feasible, consider biodegradable SUPs, especially for PS items
4	Establish defined diversion streams to teaching labs to reuse non-contaminated SUP
5	For non-reusable PP plastics, encourage donations to circular economies
6	Use waste as an asset by establishing rebate schemes with decontaminated SUPs and utilise packaging materials such as cardboard or EPS

*SUP* single use plastic, *PS* Polystyrene, *PP* Polypropylene, *EPS* expanded polystyrene (Styrofoam).

Suggestions for laboratory sustainability to be placed on an equal footing with safety and data management have been made and for this to be incorporated this into induction sessions for new team members [29]. SUP alternatives are already available for basic laboratory consumables items and compare favourably with current gold standards, with more bespoke items likely to be on the horizon. Finally, as cancer research is frequently funded by public money scientists have a duty of care to consider the environmental impact of their research and take positive steps towards sustainability and achieving net zero.

## Data Availability

Data supporting the findings of this study are available within the article.
